# IMPACT OF THE COVID-19 PANDEMIC ON EMERGENCY UPPER LIMB SURGERIES IN A QUATERNARY HOSPITAL

**DOI:** 10.1590/1413-785220243204e278237

**Published:** 2024-10-07

**Authors:** Erick Yoshio Wataya, João Pedro Teixeira Basmage, Giuliana Olivi Tanaka, Guilherme Moreira Dias, Luiz Sorrenti, Luciano Ruiz Torres, Teng Hsiang Wei, Marcelo Rosa de Rezende, Rames Mattar

**Affiliations:** 1. Universidade de Sao Paulo, Faculdade de Medicina, Hospital das Clinicas HC-FMUSP, Instituto de Ortopedia e Traumatologia, Sao Paulo, SP, Brazil

**Keywords:** COVID-19, Upper extremity, Orthopedic procedures, Elective surgical procedures, Emergency treatments, COVID-19, Membro superior, Procedimentos ortopédicos, Procedimentos cirúrgicos eletivos, Tratamento de emergência

## Abstract

The COVID-19 pandemic has triggered a global crisis in health systems worldwide. Emergency care services have been overloaded, and there have been different changes in the patient’s profile and the most frequent diagnoses. The aim of the study was to compare the number of emergency surgeries in the Hand and Microsurgery group of the quaternary hospital (IOT-FMUSP) from March 2020 to February 2022, the pandemic period, with the previous two years, March 2018 to February 2020. Two hundred and seventy-two patients were evaluated, with a mean age of 39.54 ± 17 years (range 1 to 90 years), 12.50% (n = 34) women and 87.50% (n = 238) men. Between March 2018 and February 2020, 142 (52.21%) emergency upper limb surgeries were performed; between March 2020 and February 2022, 130 surgeries were performed (47.79%). There was a reduction in upper limb surgeries in patients between 26–45 years and blunt injury surgeries. There was also an increase in surgeries in patients over 46, amputations, fractures, re-implantation procedures, and open fracture fixation. **Level of evidence III, Retrospective Comparative Study.**

## RESUMO

A pandemia por COVID-19 desencadeou uma crise global nos sistemas de saúde ao redor do mundo. Serviços de atendimento de urgência sofreram sobrecarga e diferentes mudanças no perfil do paciente atendido bem como dos diagnósticos mais frequentes. O objetivo do estudo foi comparar o número de cirurgias de urgência, no grupo de Mão e Microcirurgia, do hospital quaternário (IOT-FMUSP) ocorridos de março de 2020 a fevereiro de 2022, período pandêmico; com os dois anos anteriores, de março de 2018 a fevereiro de 2020. No total foram avaliados 272 pacientes com idade média de 39,54 ± 17 anos (variação 1 a 90 anos), sendo 12,50% (n = 34) de mulheres e 87,50 % (n = 238) de homens. Entre março de 2018 a fevereiro de 2020 foram realizadas 142 (52,21%) cirurgias de urgência em membro superior e de março de 2020 a fevereiro de 2022, 130 cirurgias foram realizadas (47,79%). Identificou-se redução do número de cirurgias em membro superior em pacientes entre 26-45 anos e do número de cirurgias por ferimentos corto contusos. Além do aumento no número de cirurgias em pacientes acima de 46 anos, número de casos de amputações, fraturas, procedimentos de reimplante e fixação por fraturas expostas. **Nível de evidência III, Estudo restrospectivo comparativo.**

## INTRODUCTION

 The COVID-19 pandemic generated an overload of health systems and services worldwide in relation to the structural component, need to allocate financial and personal resources and adaptation of safety protocols to minimize the coronavirus spread. ^1^^,^^2^ Therefore, even when faced with serious situations, patients avoided seeking health services for fear of contracting the virus in these environments. ^3^

 The demand for orthopedic care decreased during phases of greater restrictions. This reduction affected mainly the number of elective surgeries with a large number of procedures postponed or canceled, which caused possible harm to patients’ quality of life and increased the challenge of managing waiting lists. ^4^ Consequently, there was an increase in the proportion of emergency orthopedic surgeries, and the most serious cases in health services needed to be prioritized. ^5^

 Although there was a reduction in the absolute number of orthopedic consultations in the emergency setting and of the volume of surgeries performed, other services faced a real increase in demand. This was due to significant changes promoted by Brazilian states in the hospital organization, aiming to free up beds for patients with COVID-19. ^6^^,^^7^ In other words, while in some areas the activity decreased, in others there was a considerable increase in pressure on orthopedic medical services. 

 Changes in the service model also emerged during this period around the world, such as the “single service” concept. In this model, all healthcare professionals would be concentrated in the same physical space, including with the presence of a surgical arch in the plastering room, which emerged as a strategy to promote more effective care provision, reducing unnecessary patient traffic within the hospital. ^8^

 Although there are some similarities in the epidemiological profile of worldwide patients with upper limb injuries who seek emergency services, the types of accidents that result in these injuries may vary according to each country’s economic profile. In Brazil, a country considered underdeveloped, there is a predominance of injuries due to occupational accidents, traffic accidents, and domestic accidents. In developed countries, there is a predominance of injuries in sporting activities, falls, and occupational traumas. ^9^^,^^10^

 These upper limb injuries requiring urgent surgical procedures presented important changes in the epidemiological profile during the COVID-19 pandemic, as there was a reduction in the number of elective procedures ^11^ in some specialized centers across the globe. A change in the cause of injuries was also noted, with a predominance of acute traumatic injuries caused by domestic accidents, handiwork, and serious infections. ^12^

In Brazil, it is believed that the number of emergency surgeries, mainly related to severe upper limb trauma, which result in fractures, dislocations, and amputations, for example, has also decreased due to low exposure to risk factors.

 The *Hospital das Clínicas* of the *Faculdade de Medicina* [School of Medicine] of *Universidade de São Paulo* [University of São Paulo] (HC-FMUSP) is a quaternary hospital, with specialized care in trauma and complex cases. 

The objective of the study is to evaluate the COVID-19 pandemic effect on the number of urgent upper limb surgeries, performed by the Hand and Microsurgery group, and the trauma patients’ epidemiological profile taking into consideration the pre-pandemic period from March 2018 to February 2020 and the pandemic period between March 2020 and February 2022.

## METHODS

This is a cross-sectional observational study, with retrospective data collection from medical records of patients treated at the Institute of Orthopedics and Traumatology of Hospital das Clínicas of FMUSP (IOT-HC-FMUSP), a reference in the complex upper limb trauma care. This study was approved by the Research Ethics Committee (IOT-HC-FMUSP) under protocol number 4.914.423.

Patients of both sexes and of any age, with upper limb injuries that required emergency surgery, such as fractures/dislocations, neurovascular injuries, infections and amputations were included. Patients with incomplete data, data prior to the period, and with a diagnosis that did not involve the upper limb were excluded.

The clinical variables were gender, age, injury side, injury location, injury diagnosis, injury type, trauma mechanism, procedure performed in the emergency room, and need for re-approach during hospitalization. For the dependent variable, the pre-COVID-19 pandemic (patients seen between March 2018 and February 2020) and the pandemic period (patients seen between March 2020 and February 2022) were considered.

### Statistical analysis

The database was created using Excel version 2016. For statistical analyses, Stata 13.0 software (Stata Corp LP, College Station, TX, USA) was used. Statistical significance was established using a cutoff value of p < 0.05. Descriptive analyses are presented in absolute numbers (n) and relative frequencies (%), together with the mean, standard deviation, and confidence interval (95% CI). One used the Chi-square test (χ 2) or Fisher’s exact test in the bivariate analysis.

## RESULTS

The sample consisted of 272 patients with a mean age of 39.54 ± 17 years (range 1 to 90 years), with 12.50% (n = 34) of women and 87.50% (n = 238) of men. Between March 2018 and February 2020, 142 (52.21%) emergency orthopedic upper limb surgeries were performed, and between March 2020 and February 2022, 130 surgeries (47.79%).

 Additional data on the prevalence of injuries and surgeries in the pre-COVID-19 pandemic period and during the pandemic are presented in Table 1 . 

**Table 1.** Prevalence of surgeries according to epidemiological data and upper limb injury data, comparing the pre-COVID-19 pandemic period with the pandemic

**Variable**	**Total**	**Pre-pandemic period**	**Pandemic**	p
Gender				0.409
Female	34 (12.50 %)	20 (14.08 %)	14 (10.77 %)	
Male	238 (87.50 %)	122 (85.92 %)	116 (89.23 %)	
Age				**0.016**
0 to 25	59 (21.69 %)	28 (19.72 %)	31 (23.85 %)	
26 to 45	112 (41.18 %)	70 (49.30 %)	42 (32.31 %)	
46+	101 (37.13 %)	44 (30.99 %)	57 (43.85 %)	
Injury side				0.395 *
Right	113 (41.54 %)	58 (40.85 %)	55 (42.31 %)	
Left	157 (57.72 %)	84 (59.15 %)	73 (56.15 %)	
Bilateral	2 (0.74 %)	0 (0.00 %)	2 (1.54 %)	
Injury location				0.225 *
Finger	223 (81.99 %)	117 (82.39 %)	106 (81.54 %)	
Hand	31 (11.40 %)	13 (9.15 %)	18 (13.85 %)	
Wrist	4 (91.47 %)	1 (0.70 %)	3 (2.31 %)	
Forearm	8 (2.94 %)	6 (4.23 %)	2 (1.54 %)	
Arm	2 (0.74 %)	1 (0.70 %)	1 (0.77 %)	
Hand and finger	3 (1.10 %)	3 (2.11 %)	0 (0.00 %)	
Wrist and finger	1 (0.37 %)	1 (0.70 %)	0 (0.00 %)	
Injury diagnosis				0.163 *
Fracture	88 (32.35 %)	49 (34.51 %)	39 (30.00 %)	
Amputation	131 (48.16 %)	64 (45.07 %)	67 (51.54 %)	
Laceration-contusion injury	25 (9.19 %)	9 (6.34 %)	16 (12.31 %)	
Infection	9 (3.31 %)	6 (4.23 %)	3 (2.31 %)	
Necrosis	4 (1.47 %)	3 (2.11 %)	1 (0.77 %)	
Tendon injury	15 (5.51 %)	11 (7.75 %)	4 (3.08 %)	
Injury type				0.000 *
Laceration-contusion injury	135 (49.63 %)	87 (61.27 %)	48 (36.92 %)	
Crush injury	19 (6.99 %)	6 (4.23 %)	13 (10.00 %)	
Amputation	80 (29.41 %)	34 (23.94 %)	46 (35.38 %)	
Infection	13 (4.78 %)	9 (6.34 %)	4 (3.08 %)	
Fracture	22 (8.09 %)	5 (3.52 %)	17 (13.08 %)	
Tendon injury	3 (1.10 %)	1 (0.70 %)	2 (1.54 %)	
Mechanism				0.156 *
Injury	196 (72.06 %)	98 (69.01 %)	98 (75.38 %)	
Fall	42 (15.44 %)	20 (14.08 %)	22 (16.92 %)	
Bite	4 (1.47 %)	3 (2.11 %)	1 (0.77 %)	
Crush injury	25 (9.19 %)	18 (12.68 %)	7 (5.38 %)	
Ring degloving	2 (0.74 %)	2 (1.41 %)	0 (0.00 %)	
Post-operative necrosis	2 (0.74 %)	1 (0.70 %)	1 (0.77 %)	
Re-implantation attempt	1 (0.37 %)	0 (0.00 %)	1 (0.77 %)	
Procedures performed in the emergency room				**0.005**
Fixation	52 (19.12 %)	22 (15.49 %)	30 (23.08 %)	
Surgical cleaning	23 (8.46 %)	13 (9.15 %)	10 (7.69 %)	
Regularization	46 (16.91 %)	22 (15.49 %)	24 (18.46 %)	
Re-implantation	55 (20.22 %)	21 (14.79 %)	34 (26.15 %)	
Tenorrhaphy	25 (9.19 %)	13 (9.15 %)	12 (9.23 %)	
Nail bed repair	13 (4.78 %)	12 (8.45 %)	1 (0.77 %)	
Neurorrhaphy	2 (0.74 %)	1 (0.70 %)	1 (0.77 %)	
Revascularization **	40 (14.71 %)	29 (20.42 %)	11 (8.46 %)	
Flap ***	15 (5.51 %)	9 (6.34 %)	6 (4.62 %)	
Graft	1 (0.37 %)	0 (0.00 %)	1 (0.77 %)	
Need for reapproach				0.084
No	256 (94.12 %)	137 (96.48 %)	119 (91.54 %)	
Yes	16 (5.88 %)	5 (3.52 %)	11 (8.46 %)	

* Fisher’s exact test

** Revascularization + neurorrhaphy + tenorrhaphy/ Fixation + neurorrhaphy/ Tenorrhaphy + neurorrhaphy + revascularization/ Fixation + tenorrhaphy/ Regularization + neurorrhaphy + fixation + tenorrhaphy/ Nail bed repair + fixation + revascularization + regularization/ Fixation + tenorrhaphy + neurorrhaphy/ Fixation + neurorrhaphy

*** Revascularization + ulnar neurorrhaphy + tenorrhaphy / Re-implantation + tenorrhaphy + neurorrhaphy / Tenorrhaphy+ flap / Surgical cleaning + db + fixation with KW / fixation; regularization; tenorrhaphy + neurorrhaphy / Revascularization + neurorrhaphy + tenorrhaphy + fixation

There were statistical differences in the performance of surgeries during the pandemic considering age (p = 0.016), injury type (p = 0.016) and in relation to surgical procedures performed in the emergency room (p = 0.016).

There was a reduction in the number of surgeries during the pandemic in patients aged between 26 and 46 years old and an increase in the number of surgeries in patients over 46 years old.

Regarding the injury type, it is possible to observe a reduction in the number of surgeries due to laceration-contusion injuries and infections, an increase in the number of crush injuries, amputations, fractures, and tendon injuries.

Regarding surgical procedures performed in the emergency room, during the pandemic there was an increase in the number of regularizations, re-implantations and fixation/osteosynthesis of fractures.

**Figure 1.**
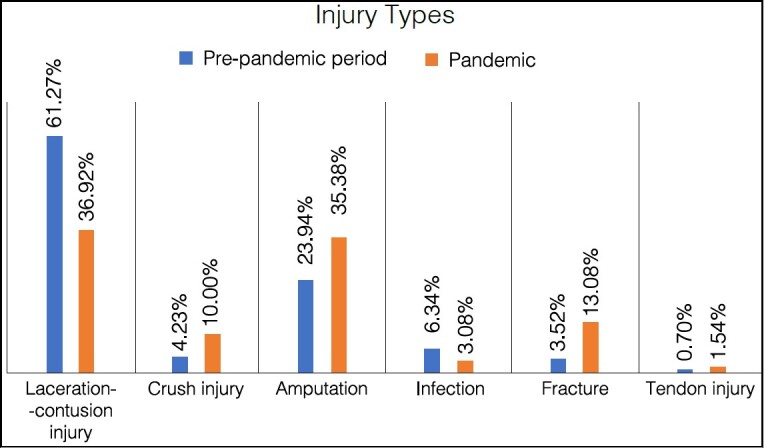
Prevalence of the type of upper limb surgical injuries comparing the pre-pandemic period with the pandemic period.

Figure 1 shows the prevalence distribution of upper limb surgeries according to the injury type comparing the pre-pandemic period with the pandemic period. (p = 0.016), with an increase in cases of crush injuries, amputations, and fractures. 

**Figure 2.**
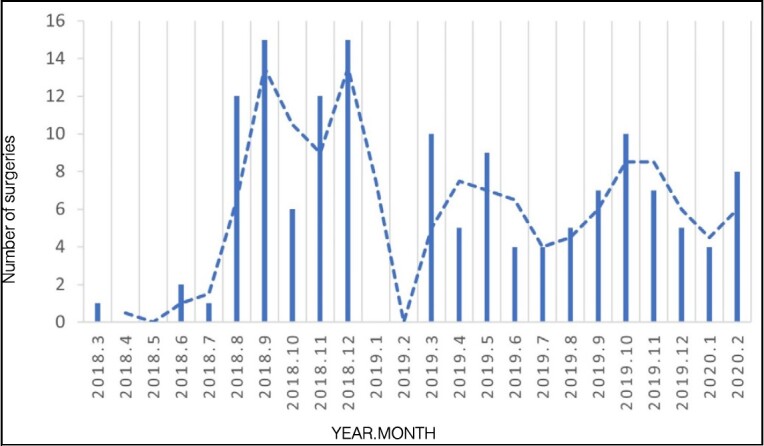
Total number of surgical procedures performed in the pre-COVID-19 pandemic period.

**Figure 3.**
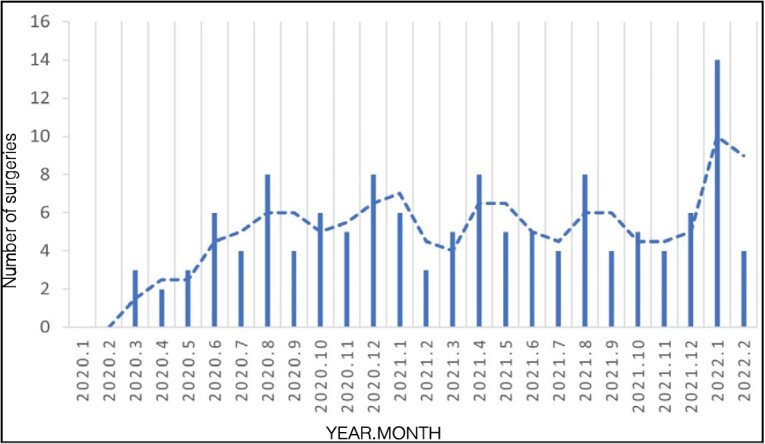
Total number of surgical procedures performed during the COVID-19 pandemic.

Figures 2 and 3 show the number of emergency surgeries month by month during the pre-pandemic period and during the pandemic period. 

## DISCUSSION

 When comparing the pre-pandemic periods with the pandemic period, a decrease in the total number of emergency orthopedic upper limb surgeries was observed. During the first period, 142 surgeries (52.21%) were performed, while in the second period there were 130 surgeries (47.79%). This reduction in the number of surgeries during the pandemic may be related to several factors, which include mobility restrictions imposed by social isolation measures, as well as greater awareness about seeking medical care only for essential cases. ^13^ Similar studies have also reported decrease in the number of surgical procedures, with values ranging from 32% to 69% in emergency sectors. ^14^^,^^15^^,^^16^

 There was a significantly higher prevalence of hand injuries in male patients during the pandemic and also before it, with ages ranging between 26 and 45 years. This trend was also identified by other studies, ^12^^,^^15^ suggesting that males are is at increased risk of suffering hand injuries, regardless of the pandemic context. 

 At the global level, services with surgical specialties have undergone a significant reduction in admissions and surgical volume. Blum et al. showed that there was a reduction not only in the number of elective consultations, but there was also a drop in the number of trauma surgeries (around 21.2% to 66.7%) and in the number of elective surgeries (33.3% to 100%) during the pandemic. ^17^ In this study, emergency procedures showed an about 4.5% reduction. Relaxation of restrictive measures in the final pandemic period and the mass population vaccination may have favored the return to work and sports, which pose a risk of new accidents in the upper limb requiring urgent surgery; besides, accidents may also happen at home. 

Regarding the injury mechanism, no significant differences were identified. Injuries following falls remained the most common injury type in both periods.

 The performance of fixation procedures continued to predominate in urgent situations, even more so during the pandemic, compatible with the period of relaxation of restrictive measures. An increase in re-implantation cases and a reduction in the need for revascularization were also observed. Lim et al., in their systematic review, found a reduction in the total number of hand injuries and fractures, but, on the other hand, noted an increase in domestic accident injuries and occupational accidents. ^18^ In this period of social isolation, the increase in the number of domestic accidents with circular saws may have led to more cases of amputations requiring re-implantation, as seen in Table 1 . 

 There was a statistically significant increase in fractures that required urgent intervention for fixation. Saleh et al. also noted an increase in the number of emergency surgeries required for cleaning, surgical debridement, and extremity fixation. ^19^

**Figure 4.**
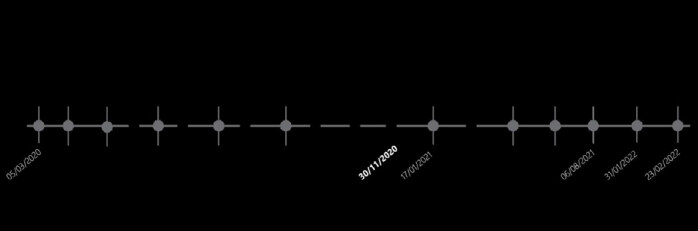
Timeline of the COVID-19 pandemic in the state where the study was conducted

 The analysis of the number of surgeries month by month during the pandemic ( Figure 3 ) in comparison with the main events that occurred ^20^ in the same period ( Figure 4 ) already demonstrates a low number of surgeries even before the World Health Organization declared the pandemic, in March 2020. With the beginning of the quarantine decreed in the state, some urgent cases occurred, but in smaller numbers than usual, perhaps due to domestic accidents. The beginning of the relaxation of restrictive measures in June 2020 coincides with an increase in emergency surgeries; patients were more exposed to risky situations at work, with a propensity to suffer injuries from circular saws and industrial machines, for example. From then on, the number of emergency surgeries remained relatively constant month by month. From December 2021 to January 2022, when a large part of the population was already vaccinated with at least one dose of the vaccine, there was a large increase in emergencies, reaching 14 cases in one month. At the end of January and beginning of February 2022, when the country again experienced a new outbreak with an increase in deaths from COVID-19, there was a new drop in emergency upper limb surgeries. 

## CONCLUSION

The pandemic reduced the use of public health services in Brazil. However, emergency care continued with changes in relation to the patient’s age, injury type and surgical procedures performed during the pandemic.

The social restriction and relaxation measures in force during the COVID-19 pandemic also influenced the number of emergency surgeries during this period.
